# Cost-Efficient Network Planning for the Cross-Border Baltic Corridor—A Study

**DOI:** 10.3390/s23198111

**Published:** 2023-09-27

**Authors:** Osama Elgarhy, Muhammad Mahtab Alam, Anet Tammets, Priit Roosipuu, Guntis Ancāns, Guntars Saidāns, Jurijs Tutovs, Klāvs Saliņš, Aleksandrs Vērdiņš, Māris Aleksandrovs, Arnis Peršēvics, Dainis Zariņš, Mart Uusmaa, Ove Uhtlik, Priit Soom

**Affiliations:** 1 Thomas Johann Seebeck Department of Electronics, Tallinn University of Technology (TalTech), Ehitajate tee 5, 19086 Tallinn, Estonia; muhammad.alam@taltech.ee (M.M.A.); priit.roosipuu@taltech.ee (P.R.); 2 “Electronic Communications Office of Latvia” SJSC, Eksporta iela 5, LV-1010 Rīga, Latvia; guntis.ancans@vases.lv (G.A.); arnis.persevics@vases.lv (A.P.);; 3 Telia Eesti AS, Mustamäe tee 3, 15033 Tallinn, Estonia; 4 Elisa Eesti AS, Sopruse pst. 145, 13425 Tallinn, Estonia; 5 Eesti Lairiba Arenduse Sihtasutus (ELASA), Narva maantee 5, 10117 Tallinn, Estonia; priit.soom@elasa.ee

**Keywords:** 5G networks, connected and automated mobility, cross-border, 5G coverage

## Abstract

Performing 5G coverage planning across borders introduces real-life challenges related to legalities, intercountry agreements, and binding documents. This work provides RF network modelling exercise results to provide uninterrupted 5G coverage to the Via Baltica and Rail Baltica transport corridors crossing Estonia and Latvia and on the border with Lithuania, as well as the Tallinn–Tartu–Valga and Valka–Valga roads (Latvia–Estonia), capable of cross-border 5G services. The study starts by identifying and interviewing stakeholders from different sectors of operation in the Baltic states and Europe and then provides an overview of some of the main legal acts and documents regulating the electronic communications sector in the Baltic states and Europe. Furthermore, 5G network requirements are proposed. In addition, the necessary and existing passive and active infrastructure is described, including spectrum management-related analysis, where the RF bands 700 MHz and 3500 MHz are analysed. Finally, coverage planning is performed. The network modelling results aim to foresee the number of new sites that need to be built on the said transport corridors, also examining the existing infrastructure for such purposes. Additionally, an estimated timeline for building the new sites is provided.

## 1. Introduction

Connected and automated mobility (CAM) services are attracting major industrial and academic interest. The European Commission, particularly the Connecting Europe Facility Programme—Digital (CEF Digital) funding program, has awarded funds to 15 projects to support cross-border 5G infrastructure deployment and open the door for CAM services. The 15 funded projects are divided into study projects and work projects. Work projects are long-term projects that are intended for infrastructure deployment. On the other hand, study projects are short-term projects that are considered as steppingstones for future projects [1]. This study is part of the “5GS: 5G CORRIDOR STUDY FOR LATVIA, ESTONIA, AND LITHUANIA” study project that aims to provide cross-border technical solutions for the deployment of 5G infrastructure along the Baltic Sea and the Baltic Rail Corridors capable of providing 5G services in the Baltic states [1,2].

In Estonia, Latvia, and Lithuania, 5G services have been launched for selected mobile operators but are mainly focusing on (and designed for) an enhanced mobile broadband (eMBB) service and covering mainly bigger cities, but not only. The availability of 5G infrastructure in transport corridors is an important step in promoting sustainable mobility, developing innovation in transport, promoting interconnected and automated driving, freight transport logistics, and improving road safety. Ensuring the continuity of services both within the transport corridor and across national borders is an essential precondition for the successful digitalization and development of the road.

The European Commission (EC) DESI document is utilized to assess a country’s readiness for 5G and overall connectivity. DESI is the digital economy and society index. DESI is an index that the EC uses to monitor the digital progress of the member states. Thus, each state achieves a given score according to its progress. The DESI 2021 Latvian report [3] states that Latvia has a score of 50.4 in terms of connectivity and ranks 14th among EU countries. However, Latvia’s score is only 29% in terms of readiness for 5G, and the country is considered lacking in terms of 5G coverage. In Latvia, experience to date has shown that previous generations of mobile communications technologies have been developed most rapidly by electronic communications operators in areas with potentially higher economic benefits, meaning that the installation of 5G base stations (BSs) takes place in those parts of Latvia where the company’s mobile network has the highest load and the concentration of simultaneous mobile network consumers. The legitimate objectives of the communication “5G for Europe: An Action Plan” and the growing reliance on seamless connectivity require national regulatory authorities to consider different approaches and conditions for the provision of comprehensive mobile coverage and the use of the relevant radio spectrum. The DESI 2021 Estonian report [4] states that Estonia’s score is higher than the average EU score, 59.4 and 50.7, respectively, and ranks seventh. The country is the leader in digital public services and has good human capital performance. Estonia is still a medium-sized provider of connectivity and still has a gap in 5G deployment. Estonia’s innovative start-ups are flourishing. The RF network planning activity in this study will provide a clear vision of the calculation results to ensure uninterrupted 5G coverage.

The performance target of network planning is the percentage of coverage area along the transport corridor for the −110 dBm and the more challenging −95 dBm signal levels. Particularly, the aim of this study is to perform full network planning to ensure 5G coverage along the Via Baltica and Rail Baltica transport corridors, crossing Estonia, Latvia, and the border of Latvia–Lithuania, as well as on the Valka–Valga road (the Tallinn–Tartu–Valga road in Estonia) for 700 MHz and 3500 MHz frequency bands. To keep the coverage planning cost-efficient, cost reduction methods are provided. Also, for the sake of cost efficiency, the current spectrum utilization and existing infrastructure are investigated and used as the basis of coverage planning; then, the new infrastructure is proposed to reach the performance targets. Therefore, the study provides first the coverage achieved using the existing infrastructure and then the coverage due to the proposed new infrastructure, including the locations of the new sites for such infrastructure.

Furthermore, the study presents aspects that should be considered in real-life coverage planning other than the ones considered for theoretical ones, especially in a cross-border scenario. First, stakeholders, such as governmental bodies and mobile network operators, make the decisions to adopt new technologies and network upgrades according to considerations such as laws, finance, market readiness, or capabilities. Thus, these stakeholders are key components of any network planning. Second, in a cross-border scenario, the degree of cooperation and cross-border planning is governed by law and internationally binding documents. Third, the spectrum assignment to the countries across the border should be identified to have successful cross-border coverage. Fourth, the existing infrastructure should be identified and utilized to the fullest; otherwise, operators will incur extra costs. Finally, the requirements, either identified by the stakeholders or the use cases, should be identified. Thus, in this study, before performing network coverage planning, we start by identifying and interviewing the stakeholders and reporting the binding documents; then, we report the main requirements, to give an overview of real-life cross-border network planning. 

In summary, this study is part of the first wave of projects selected by the EC for the transport corridor’s 5G coverage. Working on the transport corridor comes with more challenges than intracountry 5G network planning because other than the conventional network planning technical challenges, intercountry cooperation and organization are needed. Moreover, operators are limited by intercountry coverage and radiation regulations while targeting seamless service continuity and roaming. This study provides the main steps for the cross-border transport corridor 5G coverage planning process, in addition to presenting the expected average cost, timeline, and needed infrastructure. Additionally, it conveys the actual mobile network operators’ (MNOs’) network planning point of view, tools, and procedures. The planning is conducted for both road and rail corridors. On one hand, this study can be replicated in other transport corridors because the main steps and challenges are the same. On the other hand, some of the specifics of the obtained network planning results are unique to the studied transport protocol. For example, the antenna height is chosen based on the geographical nature of the countries. Also, the final cost depends on the existing infrastructure and the prices within the studied countries. Therefore, this study, which is the first of its kind (to the best of our knowledge), can serve as a blueprint for 5G transport corridor coverage planning. This study presents a holistic overview of network planning that includes introducing the stakeholders, identifying binding documents, and defining the requirements, in addition to providing the network planning for the transport corridor, including the number of needed sites, used spectrums and signal levels, costs, and timeline. 

The remainder of this paper is organized as follows. Section 2 is dedicated to binding documents and stakeholders’ interviews. The network requirements for the coverage planning are presented in Section 3. Section 4 presents the network planning preliminaries, i.e., the existing infrastructure, cost reduction methods, the exact location of the 5G cross-border corridor, and planning parameters and tools. The results of the network planning are presented in Section 5. Finally, the conclusions and recommendations are presented in Section 6.

## 2. Phase 1—Stakeholders and Binding Documents

This section presents, not exclusively, stakeholders’ general views and necessities of 5G network planning and deployment in the respective areas and regulatory documents.

### 2.1. Stakeholders

There is often a gap or delay between theoretical advances and what is offered in real life. One of the main factors is the ability of the stakeholders and key players in the market to adopt these new technologies. Thus, in this part of the work, stakeholders from various sectors in the Baltic states and Europe were identified and interviewed. The study included as many stakeholders as possible within the available timeframe for the study. The aim is to aid the implementation of stable 5G coverage along the Via Baltica, Rail Baltica, the Tartu road (Tallinn–Tartu–Valga (EST)), and Valka–Valga (LAT) transport corridors in the Baltic states.

Examples of the interviewed stakeholders are state administrative institutions (including state and local government-controlled and financed capital companies), electricity suppliers, telecommunications companies, and the transport industry. It is to be noted that fewer interviews were conducted with Lithuania’s stakeholders because the National Roadmap for 2021–2030 of Lithuania foresees making efforts to improve Lithuania’s digital connectivity, and one of the planned activities is to ensure the earliest possible deployment of next-generation mobile 5G technology. Consequently, in 2021, the Ministry of Transport and Communications of the Republic of Lithuania commissioned a feasibility study [5] of EU investments in the development of high-speed communications in Lithuania. Therefore, Lithuania can share its know-how through the Ministry of Transport and Communications of the Republic of Lithuania.

The list of the interviewed stakeholders can be divided into the following:

- Ministries and state administrative institutions (including state and local government-controlled and financed capital companies);
- Mobile network operators from involved countries;
- The transport industry;
- Electricity suppliers;
- The communication equipment manufacturing industry;
- Autonomous car manufacturers.

The interview questions were grouped into several thematic blocks that covered topics such as the following:

- Binding/regulatory/strategic documents;
- Regulatory acts related to 5G mobile communication networks near transport corridors;
- The process of necessary permit acquisition;
- The exact locations of the studied 5G corridors;
- The potential locations of 5G BSs;
- Previous/current/future projects related to 5G corridors in the Baltic states;
- Use cases and requirements for the 5G mobile communication network in the field of transport;
- Requirements and mechanisms of mobile communication networks when crossing national borders (if any);
- Certification and monitoring of 5G mobile communication networks in the world;
- Planning aspects of 5G networks and technical parameters of 5G mobile communication networks;
- Infrastructure sharing possibilities;
- Other relevant topics.

The obtained information led to the identification of factors that would promote the development of more successful 5G mobile communications coverage near the respective transport corridors and to understand in more detail the problems related to 5G coverage along the transport corridors. Some examples of some of the main issues mentioned during the interviews, which should be considered while planning a successful rollout of 5G mobile coverage, are the following:

- Information used for network planning (MNOs): MNOs have provided information that is used in this study for coverage planning.
  ○ The spectrum assignment and cooperation on the corridor: Knowing the spectrum utilization in each country is essential for coverage planning. For example, the coverage area is influenced by the used spectrum. Some of the obtained information about the spectrum and cross-border cooperation is provided in Section 4.3.
  ○ The key required performance metrics: The MNOs must deliver a certain quality of service, as will be presented in Section 3.4, governed by a set of performance metrics. An example of these performance metrics is presented in Section 3.3.
  ○ Design parameters: There is a set of coverage planning tools used by the MNOs. Certain design parameters are entered into these tools for coverage planning and simulations. An example of these design parameters is presented in Section 4.5.
  ○ Cost-effective infrastructure parameters: Some parameters are obtained through real-life consideration and knowledge of the network behaviour in the specific geographical area. These parameters are based on the MNOs’ experience and knowledge and affect the cost of the design. An example of these parameters is the tower heights used in this study.
- Stakeholders such as state administrative institutions and telecommunications companies mentioned two types of requirements and considerations.
  ○ National considerations: There is more than one MNO in one country. Building new sites and passive infrastructure is one of the most cost-consuming elements of coverage planning; see Section 5.4. The state provides rent-free land for this reason; see Section 4.6.1; additionally, MNOs often share sites. Examples of national considerations are as follows:
    ▪ Terms and conditions for shared use of towers;
    ▪ Construction of passive infrastructure at the national level.
  ○ Intercountry considerations: For cross-border coverage planning, different stakeholders, e.g., state administrative institutions and MNOs in both countries, should communicate and cooperate to reach unified rulemaking and regulations. Examples of the raised intercountry considerations are as follows:
    ▪ Communication between stakeholders;
    ▪ Unified rulemaking;
    ▪ Development of unified regulations for cross-border locations.
- Some stakeholders raised questions, to be prepared for future requirements and challenges. Future services might have different or more stringent requirements than the current ones. What are these requirements? And is the current spectrum assignment suitable for these requirements? Examples of the questions are as follows:
  ○ What will be the necessary services of the future?
  ○ Will the 700 MHZ and 3500 MHz radio frequency bands be sufficient for future services?

### 2.2. Binding Documents Related to 5G Corridors

The legal framework for the EU’s approval of automated vehicles is still in its infancy, as it is a complex problem that not only affects the physical and digital infrastructure of automobiles and telecommunications but also involves communication technologies and cooperation between different ecosystems. However, the current EU legislation is suitable for being placed on the automated and connected vehicles market.

The binding documents for Via Baltica and Rail Baltica:

- In September 2018, in Riga at the 5G Techritory forum, Lithuania, Estonia, and Latvia signed a Memorandum of Understanding (MoU) and agreed to cooperate to deploy a 4G and 5G network along a section of the Via Baltica, Kaunas (Lithuania)–Riga (Latvia)–Tallinn (Estonia)–Lithuanian/Polish border, to test autonomous vehicles, to promote innovation and sustainable mobility in transportation systems, and to increase road safety.
- In September 2020, Estonia, Lithuania, Poland, and Latvia signed a Memorandum of Cooperation for the introduction of solutions for 5G and connected and automated mobility (CAM). The signed memorandum approved the road map for the construction of 5G and CAM corridors along the Baltica road connecting the four countries. Indeed, 5G networks are an important stimulant for innovative transport solutions and improving traffic safety in the North Sea–Baltic shipping corridor. In line with EU goals for 5G availability on large land transport routes, the right to use 700 MHz spectrum bands will be one condition for providing 5G coverage on large land transport roads under the Trans-European Transport Network (TEN-T). The Baltic Expert Working Group is working on the question of providing a passive broadband infrastructure for the Baltic Via (E67) and is identifying the location of the existing fibre-optic cable network along the Latvian section of the Baltic Via (E67) motorway, while identifying the mobile BSs coverage and the availability of electricity infrastructure. In addition to binding documents, strategies and legal acts are enforced in each country to drive the development of and to regulate telecommunication networks, such as in [6,7,8,9,10,11,12,13,14,15,16,17,18,19,20].

## 3. Phase 2—Requirements and Infrastructure

In order to perform network planning, we need first to identify the network requirements and the needed infrastructure. In this section, the existing standards and requirements are analysed with the aim of obtaining initial information for the creation of relevant infrastructure for 5G corridors. There are different types of requirements; they are divided into performance, infrastructure, service continuity, and supervisory requirements. In this section, the main elements that constitute these requirements are presented.

### 3.1. Performance Requirements—Intelligent Transport Systems

One of the main goals of designing a communication network is to achieve the main performance requirements. In this section, the performance requirements of the 5G communication network over the road and rail transport corridor are discussed. This subsection starts by introducing intelligent transportation systems since covering the transport corridors falls under the umbrella of intelligent transportation systems. Next, the 5G performance requirements, including those for vehicle-to-everything (V2X) communication, are presented. Finally, an introduction to the rail communication systems along with their performance requirements is presented.

A major target for a 5G-covered transport corridor is high-quality connectivity, which can guarantee the continuity of business across the corridor and could satisfy the requirements for multi-application/service 5G services and the service requirements for rail/road operations’ safety, e.g., the Future Rail Mobile Communication System (FRMCS) and ITS. Actual network planning will make use of at least one, but most likely several, 5G bands (26 GHz, 3.6 GHz, 700 MHz, etc.), and if possible, the 1900 MHz and 900 MHz bands of FRMCS and ITS’s 5.9 GHz band.

ITS refers to measures to collect, store, and provide traffic information, in real time, in order to provide convenient safe transportation, reduce energy consumption, and maximize utilization efficiency by incorporating advanced telecommunication information and electronics technologies into automobiles and goods.

ITS has the potential to greatly contribute to a safer, cleaner, and more efficient transportation system. As a result, various regulatory and legislative initiatives in Europe have focused on ITS. The European Commission has established a legal framework to facilitate the deployment of these new transportation technologies across Europe. Furthermore, the European Commission has asked the European standards bodies to create and implement standards in support of this framework. Not unexpectedly, the standards bodies ETSI, CEN, and CENELEC are very active in this field [21].

It is worth mentioning that ITS services’ connectivity can be either to remote platforms, e.g., service providers, or to the surrounding elements, e.g., other vehicles or roadside units [22].

There are three main 5G services: eMBB, URLLC, and mMTC. Each ITS target application will belong to one of these services or be a mix of them. Additionally, some of these applications will depend on whether it is a road scenario or a rail one. Examples of these applications are as follows:

- Advanced driving: For safe traveling, efficient traffic management, and avoiding collisions. In advanced driving, the vehicle shares information. Moreover, the vehicles can be fully automated or semiautomated.
- Vehicles platooning: a dynamic group of vehicles are traveling together.
- Extended sensors: road units, pedestrians’ devices, and vehicles share their obtained sensors’ data.
- Remote driving: This is applied to either road or rail. The vehicle or the train is operated by a remote driver.
- European Train Control System (ETCS): The train sends information messages containing its speed, position, etc. The train receives supervision messages, such as movement authority permission messages.
- Automatic train operation (ATO): for the automation of the train.

#### 3.1.1. V2X and 5G Network Requirements

A specific use case of 5G systems is a 5G corridor, and some of its main characteristics are highly variable data rates and high user mobility and density. In transport corridors, issues related to traffic safety are essential, which impose stringent requirements on speed, communication range, latency, and reliability. The use of road infrastructure and vehicle sensor data is crucial for ensuring traffic safety in transport corridors. ITSs encompass a wide range of communication-related applications intended to improve traffic management, reduce impact on the environment, and improve travel safety. Roadside infrastructure is connected wirelessly to control centres for control and management purposes. The communication between the control centre and roadside infrastructure requires high-capacity connections, high service availability, and low latency for the reliable distribution of data. 

The common service requirements for 5G systems regarding the use of transport are defined in ETSI’s “Service requirements for the 5G systems” [23]. 

The requirements for high traffic density and data rate scenarios are as follows:

- Highspeed train (up to 500 km/h).
  ○ DL data rate 50 Mbps;
  ○ UL data rate 25 Mbps.
- Highspeed vehicle (up to 250 km/h).
  ○ DL data rate 50 Mbps;
  ○ UL data rate 25 Mbps.

Another key requirement is end-to-end latency. End-to-end latency depends on the target scenario. Moreover, what are particularly important for ITS services and 5G corridors are the V2X communications mode requirements. In that case, 5G requirements depend on the level of automation: no automation, full automation, or any degree in between. In the following, the required maximum end-to-end latency of four of the main V2X scenarios is presented.

- Vehicles platooning.
  ○ Low/high degree of automation: 20 ms.
- Advanced driving.
  ○ Low/high degree of automation: 100 ms.
- Extended sensors.
  ○ Low degree of automation: 100 ms;
  ○ High degree of automation: 50 ms.
- Remote driving: 5 ms.

#### 3.1.2. Future Railway Mobile Communication System

The FRMCS is a telecommunication system that is the successor of GSM-R and is designed by the UIC, in cooperation with the various stakeholders from the rail transportation sector. Moreover, it is a main enabler for rail digitalization [24].

The FRMCS will employ 3GPP transport to communicate with train users. It will eventually be similar to GSM-R and will be able to give communication features that GSM-R cannot. Multimedia communication, shorter data latencies, higher data rates, and increased communication reliability will be provided. The FRMCS takes into account end-to-end use scenarios and includes requirements that may or may not be covered by existing 3GPP specifications. Interworking requirements between the FRMCS and old communication systems are considered to assist a smooth migration from traditional communication systems (e.g., GSM) to the FRMCS.

The FRMCS will connect to the application domain using access technologies such as 3GPP radio access or others. It provides highly reliable and low-latency video and data services in a rail environment characterized by high speeds. Some of its main features and advantages are the following:

- High-reliability and low-latency video and data service;
- Rail location-based addressing, functional addressing, and emergency call;
- Emergency group communication;
- Services for train maintenance, management, control, and management for safe operation;
- Interworking with the legacy GSM-R system [25].

As for the rail scenarios and use cases, there are use cases different from the use cases of the V2X. In the following, the maximum end-to-end latency and the corresponding rail use case are presented.

- ATO: 100 ms;
- ETCS: 100 ms;
- Remote driving: 10 ms.

In addition to the latency, there are data rate and reliability requirements. Depending on the type of message, the data rate can be up to 100 Kbps, 10 Kbps, and 7 Mbps for ATO, ETCS, and remote driving, respectively. The reliability can be up to 99.9% for ATO and 99.9999% for ETCS and remote driving. 

### 3.2. Infrastructure Requirements for Building 5G Corridors

The relevant 5G corridor infrastructure consists of passive and active infrastructure. Passive infrastructure includes communication masts and communication lines for data flow to/from the core network and power supply. Communication lines can be optical networks or microwave lines. The data transmission rate on microwave lines depends on the used bandwidth. Existing microwave line equipment is capable of providing up to 3.5 Gbps using a transmission bandwidth of 500 MHz. The usable frequency resource is limited; therefore, in order to ensure the necessary amount of data transmission in the 5G corridor, the passive infrastructure must be provided an optical network connection. The active infrastructure consists of the core network and radio access network with transmitting/receiving equipment and antennas that ensure wireless data transmission to user equipment. Active infrastructure must provide signal quality parameters in the 5G corridor in accordance with the defined requirement—low latency, high data rate, high communication service availability, and high-capacity connections for the reliable distribution of data.

### 3.3. Service Continuation across Border—Roaming and Networks Handover

Since the 5G transport corridors cross the borders of several countries, it is necessary to ensure business continuity for infrastructure and users. The right to utilize the 3.5 GHz and 700 MHz spectrum bands is contingent on using them for 5G coverage along TEN-T highways. A key component is to establish cross-border collaboration so that the systems put in place can operate freely, both at the border and along the transportation corridor.

Cross-border frequency coordination agreements will govern the use of frequencies in the border area. The different agreements between countries across the border determine the rules of coordination and the usage of frequencies across the border, to limit interference and coordinate BSs installation [26]. 

Within the scope of this work, two stages of border crossing can be identified: EE–LVA and LVA–LT (including railway and road infrastructure), where the location of existing fibre-optic cable networks and the coverage of mobile BSs must be known to ensure uninterrupted service. The most important factors for end users are roaming and networks handover across borders.

- In cross-border scenarios, roaming is considered the number one service disruption factor. Improving roaming in 5G should result in seamless network changes during border crossing. Roaming can be considered as extending the service area of the user’s home network. Moreover, in CAM applications, roaming must ensure service continuation in cross-border scenarios. An additional requirement in the EU is that the communication cost, i.e., the service price, should be the same in both the home country and the visiting country.

The Council of the EU and the European Parliament (regulation 2022/612), dictates the following on roaming (6 April 2022):

Within the EU, across-the-border handover delays should be minimized by the different mobile network operators [27].

- Handover is the time in which the data transmission and receiving of a mobile device is interrupted when it moves from the serving cell to a different cell. The 3GPP put forth a great effort to minimize this time as much as possible, and the main challenge is to make it in the order of milliseconds. However, currently in 5G networks, this very brief handover time is only possible in several scenarios, such as changing the serving beam of the mobile device within the same cell. For the cross-border scenario, the most critical type of handover is the one where the mobile device has to change MNOs between two countries, i.e., inter-PLMN.

Some of the important performance indicators are presented in Table 1, where 5G KPIs related to service continuation across the border are presented.

#### Inter-PLMN Handover

The existing network handover scenarios at internal EU border crossings result in connection interruptions. Long interruptions (up to minutes) in CAM services can affect operation and safety for automotive driving during roaming. When a mobile device crosses a border and is served by the neighbouring MNO and its PLMN, management procedures should be implemented and able to support CAM functionality during an inter-PLMN handover of the service. The execution of a low-latency, smooth inter-PLMN HO (public land mobile network handover), when crossing borders, is likely the most important factor for providing minimal latency and service continuity in CAM applications. At the borders, the conditions of radio coverage notably affect inter-PLMN HO.

The inter-PLMN HO interruption time depends on several factors, such as the technologies used and the agreements across the border. However, ongoing research aims to reduce this interruption time [28]. Notably, research and large-scale trials were carried out by the 5GPPP project 5GCroCo to ensure that seamless service continuity traversing borders can be guaranteed in 5G networks. The project implementers concluded that it is technically feasible to have service interruption when crossing a border of about 120 ms, allowing the continuity of CAM services [29].

### 3.4. Supervisory Requirements

Supervision and certifications are needed to guarantee a certain quality of service and performance requirements. Certification is an important part of ensuring the quality and reliability of 5G networks. This process involves testing and verifying that the networks meet certain technical standards and specifications. Certification can be carried out by independent third-party organizations or by government regulatory bodies.

The supervision of 5G corridors is also important for ensuring that the networks operate safely and securely. Governments and regulatory bodies can set rules and guidelines for the operation of 5G networks, monitor compliance with these rules, and enforce penalties for noncompliance.

Some points crystallized after interviews with stakeholders are as follows:

- The quality of a 5G network is surveyed daily with network level key performance indicators (KPIs). There are specific KPI targets that networks should meet.
- FRMCS—the standard is still under development. It is mentioned in 3GPP releases 15, 17, 18, and 19.

#### Certification and Supervision Technical Parameters

Stakeholder engagement leads to a conclusion that the following technical parameters should be measured and monitored in 5G services along transport networks (5G corridors):

- Availability (%);
- Reference signal received power (SS-RSRP) (dBm);
- Reference signal received quality (SS-RSRQ) (dB);
- Signal-to-interference-and-noise ratio (SS-SINR) (dB);
- Throughput—user/service data rates (download speed, upload speed) (Mbps);
- Latency (end-to-end)—delay in real-time application (ms);
- Coverage (%);
- Reliability (%).

The collection and measurement of SS-RSRP, SS-RSRQ, and SS-SINR parameters, where SS stands for synchronization signal, helps to assess the coverage percentage and service availability percentage of 5G MNOs in 5G road and rail corridors. Throughput and latency can be evaluated according to specific use case requirements. In addition to the analysis and evaluation of throughput problems in the 5G coverage corridor, it will also be necessary to measure signal CSI-RSRP—CSI reference signal received power (dBm)—and CSI-SINR—CSI signal-to-noise-and-interference ratio (dB).

The 5G NR BS signal quality parameters are SS-RSRP, SS-RSRQ, and SS-SINR [30]. Briefly, these parameters are described as follows:

- SS—RSRP: These are cell-specific synchronization signals that are key parameters for handover and cell selection and can be used to compare the signal strength of the cells in the network.
- SS—RSRQ: This is an indicator of the radio link quality. Similar to RSRP, it is used for handover and cell selection; however, it includes interference information and is particularly useful at the border of the cell.
- SS—SINR: The average signal power [W] divided by the average interference and noise powers (W) over all the synchronization signal resource elements.
- CSI-RSRP and CSI-SINR: These parameters are similar to SS-RSRP and SS-SINR; however, CSI is used rather than SS.

Note that SS-RSRP, SS-RSRQ, SS-SINR, CSI-RSRP, and CSI-SINR are similar to their LTE counterparts, e.g., RSRP and RSRQ, with the difference that in LTE the reference signal is used, while in 5G the channel state information and synchronization signals are used. 

The following technical parameters could be considered as certification and supervision parameters of 5G corridors (see Table 2).

During the testing period, the worst-case scenario of the 5G network should be applied or a methodology should be developed for each case scenario. Certification measurements should be entrusted to a competent state institution that has the appropriate equipment and experience in public mobile network signal quality measurements and radio interference search.

To provide the necessary conditions for 5G covered corridor supervision, a service centre should be established which collects and processes information about compliance with the 5G corridor network requirements. The information is obtained in real time from the users of the 5G corridors.

## 4. Phase 3—Network Planning Preliminaries

After identifying the requirements for network planning, both technical and nontechnical, in this section, we prepare for the network planning phase. We start by identifying the exact location of the 5G cross-border corridor, the existing infrastructure, the spectrum, and the base station locations. Since one of the main goals of this study is to be cost-efficient, the existing infrastructure is fully identified (to be utilized), in addition to using information extracted from the stakeholders’ interviews and cost reduction methods. Thus, this section is considered an important and integrated part of network planning.

### 4.1. Definition of Exact Locations of 5G Corridors Including Cross-Border Sections via Baltica and Rail Baltica

This section provides information about the exact locations of 5G road and railway corridors in Estonia, Latvia, and Lithuania within the study. Data on the 5G road and railway corridors in Estonia, Latvia, and Lithuania were collected during the stakeholder engagement phase.

#### 4.1.1. Locations of 5G Road Corridors in Estonia, Latvia, and Lithuania

**Figure 1:**
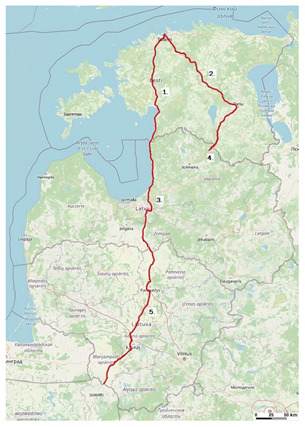
Exact locations of 5G-covered road corridors in Estonia, Latvia, and Lithuania.

Figure 1 presents the exact locations of 5G road corridors in Estonia, Latvia, and Lithuania within the study.

Each pinpoint in the figure represents the following:

1. The 5G corridor along the Via Baltica road in Estonia: Tallinn (Viru Square)–Pärnu–Ikla (Latvia border) (part of the road E67).
2. The 5G corridor along the Tartu road in Estonia: Tallinn (Viru Square)–Tartu–Valga (Latvia border) (part of the road E263).
3. The 5G corridor along the Via Baltica road in Latvia: Ainaži (Estonia border)–Riga ring road–Bauska–Grenctāle (Latvia border) (part of the road E67).
4. The 5G corridor along the Valga–Valka road in Latvia: Valga (Estonia border)–Valka (10 km of E264 road from Estonia border into Latvia side).
5. The 5G corridor along the Via Baltica road in Lithuania: Saločiai (Latvia border)–Panevėžys–Kaunas–Marijampolė (Poland border) (part of the road E67).

The Via Baltica route is a part of the international E road network—route E67. This numbering system for European roads was developed by the UN Economic Commission for Europe (UNECE).

At the same time, the Via Baltica route is part of the core network of TEN-T. The requirements for the TEN-T network are defined in Regulation (EU) No. 11 of December 2013 of the European Parliament and Council, 1315/2013, on Union guidelines for the development of the European transport network and which repealed Decision No. 661/2010/EU. According to this regulation, by the end of 2030, the roads of the TEN-T core network, including Via Baltica, must be rebuilt into high-speed roads.

In accordance with the “National Highway Strategy 2020–2040” discussed at the meeting of the Cabinet of Ministers of the Republic of Latvia on 19 June 2021, the Via Baltica route is planned to be developed until 2040, envisioning it as a two-lane high-speed road.

Globally, the Via Baltica route in the territory of Latvia will not change, but there are certain places where potential changes are possible. Smaller-scale changes (small traffic safety improvement projects) or the construction of additional lanes cannot yet be accurately predicted.

#### 4.1.2. Locations of 5G Railway Corridors in Estonia, Latvia, and Lithuania

**Figure 2:**
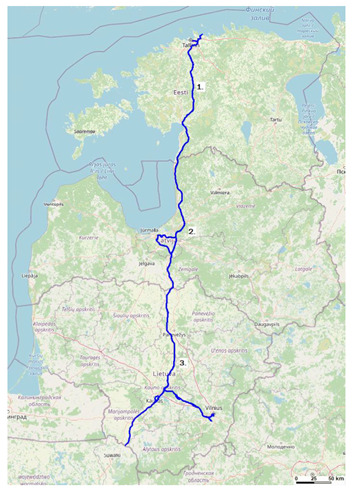
Exact locations of 5G railway corridors (Rail Baltica) in Estonia, Latvia, and Lithuania.

Figure 2 presents the exact locations of 5G railway corridors (Rail Baltica) in Estonia, Latvia, and Lithuania

Each pinpoint in the figure represents the following:

1. The 5G corridor along Rail Baltica in Estonia: Tallinn–Pärnu–near to Ikla (Latvia border).
2. The 5G corridor along Rail Baltica in Latvia: near to Ainaži (Estonia border)–Rīga–near to Grenctāle (Lithuania border).
3. The 5G corridor along Rail Baltica in Lithuania: Kiemėnai (Latvia border)–Panevežys–Kaunas–Vilnius–Marijampolė (Poland border).

### 4.2. Existing (Constructed) Passive Infrastructure

During the study, an assessment of the existing infrastructure of MNOs was carried out, as well as the possible connections of the optical network. At the time of study preparation, 5G is deployed mainly in larger cities and populated areas, but not only, in all three Baltic states. In Estonia, Latvia, and Lithuania are developed 4G networks with appropriate BSs. This passive infrastructure for networks (BS towers) can be used in the creation of planned 5G corridors.

#### 4.2.1. Existing Estonian Infrastructure

**Figure 3:**
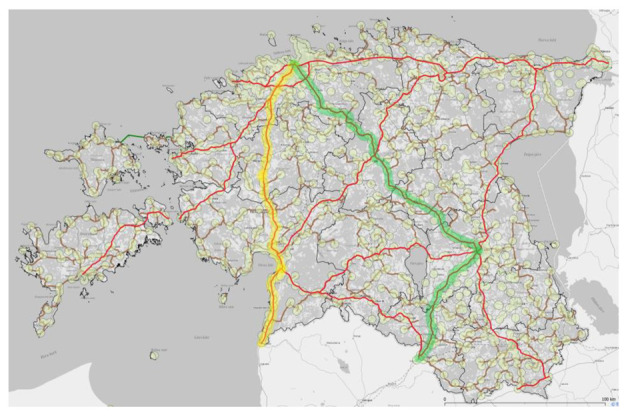
Location of BSs along the transport routes in Estonia.

In Estonia, the study examines two TEN-T roads: Tallinn–Parnu–Ikla and Tallinn–Tartu–Valga. In Figure 3, the route Tallinn–Parnu–Ikla is marked in yellow, while the route Tallinn–Tartu–Valga is marked in green. Existing BSs are marked with circles on the map (Figure 3).

Currently, the main concern for operators regarding the placement of BSs is the population density in specific places; therefore, the special placement of BSs to create special transport corridors has not been carried out.

In Estonia, a preliminary analysis of the passive infrastructure of the existing BSs has been carried out, and it has been identified that to cover the Tallinn–Parnu–Ikla route with uninterrupted 5G coverage, it is necessary to build 16 new towers. On the Tallinn–Tartu–Valga route, it is necessary to build 28 new towers for the creation of passive infrastructure for uninterrupted 5G coverage. This preliminary study did not analyse the situation with existing towers—whether they have sufficient capacity for a 5G RAN. Optical connections to BSs for the creation of a communication network have mainly been used in cities and settlements with adequate population density. Where the data transmission capacity is lower, microwave lines are used; however, their deployment is a rare-case scenario, and their deployment among MNOs has been decreasing.

#### 4.2.2. Existing Latvian Infrastructure 

In Latvia, the study examines two road transport corridors: Ainaži–Grenctāle (Via Baltica), which is a TEN-T route, and the Valka–Valga road. The BS located along the Via Baltica road Ainaži–Grenctāle and the Valka–Valga road have been identified in Latvia. 

In Latvia, an analysis of the passive infrastructure of the existing BSs has been carried out, and to build a 5G corridor along the Via Baltica route, it was concluded that it was necessary to build 15 new towers. The communication network between the appropriate BSs is established mainly using microwave lines, which are planned to be replaced with optical connection [31]. 

In Latvia, the operator neutral optical network belongs to LVRTC; additionally, there are also other owners’ optical networks (ownership and information on Latvian optical networks’ geographical extension is fragmented and is not publicly available).

#### 4.2.3. Situation in Lithuania

The project “The development of 5G network infrastructure along the trans-European transport network’s’ (TEN-T) via Baltica and Rail Baltica” has been prepared in Lithuania. This project analyses the situation of the creation of 5G corridors in the sections of Via Baltica and Rail Baltica in Lithuania. “White spots” have been identified in the project, and it has been established that to ensure the creation of a 5G corridor along the Via Baltica in Lithuania, it is necessary to build an additional 32 masts and create the appropriate communication infrastructure using an optical cable connection. In the Lithuanian project, it is planned to install 30 m high masts. The project also includes a further development concept, which provides for the installation of 18 additional masts.

The route of the Baltic Railway is planned through sparsely populated areas, so the project plans to build its own autonomous 5G infrastructure, which includes both the construction of communication towers and the creation of an optical network, electricity lines. This infrastructure is oriented towards essential needs for railway transport (the replacement of GSM-R with FRMCS). The project plans to build communication towers and offer them to operators for providing public communications. Using the passive infrastructure built by Rail Baltica, operators will be able to provide public communication needs.

### 4.3. Spectrum Utilization of Each Country and Cross-Border Coordination 

A spectrum is the basis for the deployment of wireless communication systems, and for MNOs to be able to successfully expand their network coverage and increase their capacity, sufficient spectrum resources must be available. At the EU level, three spectrum bands (the so-called pioneer bands) have been identified for the deployment of 5G (700 MHz, 3.5 GHz, and 26 GHz bands).

V2N communication for CAM services would use frequencies such as 3.5 GHz and 700 MHz, while 5.9 GHz would be utilized for V2P, V2V, and V2I short-range communications [32].

Many licensed and unlicensed bands for normal operation with a 5G network will be supported by 5G devices; however, when it comes to the PC5 interface (device-to-device communication), the options are more limited. According to the 3GPP TR 38.886 V16.3.0 (March 2021) technical report, NR V2X communication for the PC5 interface is designed to operate in such operating bands in FR1 (see Table 3).

In the study for coverage planning along the road and railway 5G corridors, 700 MHz and 3.5 GHz frequency bands are used. Information about radio frequency spectrum assignments in the 700 MHz and 3400–3800 MHz bands in Estonia, Latvia, and Lithuania is provided in the following Section 4.3.1, Section 4.3.2 and Section 4.3.3.

#### 4.3.1. Spectrum Assignment for the Frequency Band 700 MHz

This section provides information about radio frequency spectrum assignments to MNOs in the 700 MHz band (FDD and SDL frequency arrangement) in Estonia, Latvia, and Lithuania [34]. Estonia has three operators that occupy a frequency range from 703 MHz to 733 MHz in the uplink and from 758 MHz to 788 MHz in the downlink. Latvia has three operators that occupy a frequency range from 703 MHz to 733 MHz in the uplink and from 738 MHz to 788 MHz in the downlink. Finally, Lithuania has three operators that occupy a frequency range from 713 MHz to 733 MHz in the uplink and from 768 MHz to 788 MHz in the downlink.

#### 4.3.2. Spectrum Assignment for the Frequency Band 3400–3800 MHz

This subsection provides information about radio frequency spectrum assignments of MNOs in the 3400–3800 MHz band (TDD frequency arrangement) in Estonia, Latvia, and Lithuania [34]. All three countries have three MNOs; all MNOs use TDD and occupy the frequency band 3400–3800 MHz. 

In Estonia, for 3400–3800 MHz (C-band), currently in use is the secondary frequency allocation plan (60 MHz + 70 MHz or 70 MHz + 60 MHz per operator). After a few years, it would be possible to switch to primary frequency allocation, which denotes a continuous 130 MHz per operator (if all operators and regulators jointly agreed to this). As for Latvia, Telia Latvija is assigned 28 MHz (national coverage except Riga) and UNISTARS is assigned 50 MHz (for use in Riga) radio frequency spectrum rights of use in the 3600–3650 MHz frequency band.

#### 4.3.3. Cross-Border Spectrum Coordination

In border areas, the radio frequency spectrum usage conditions are regulated also by international radio frequency cross-border agreements.

For the 3400–3800 MHz band, which has the TDD frequency arrangement, it is important to have TDD networks synchronization. The synchronised TDD networks allow us to use the 3400–3800 MHz band spectrum more efficiently without applying guard bands for adjacent channels and allows us to use radio channels with higher field strength levels at the border. The synchronization of TDD networks in border areas in this frequency band is recommended, as it ensures the efficient utilization of the spectrum, particularly for outdoor deployments. Furthermore, to avoid downlink/uplink simultaneous transmissions, synchronization for crossing borders requires the use of a compatible frame structure and common phase clock.

The identified frequency bands for FMRCS services of Rail Baltica are the 900 MHz (sub-band of it) and 1900 MHz bands, which are specific to railway transport and therefore cannot be used for public purposes. The FRMCS frequency bands are the 874–880 MHz and 919–925 MHz (paired) and the 1900–1910 MHz (unpaired) bands, in accordance with ECC Decision (20)02.

This study considers the following SS-RSRP level (downlink) values: min. value −110 dBm and upgrade value −95 dBm. In Table 4, the SS-RSRP values are compared with the frequency coordination threshold values (at the border) of existing agreements concluded between Latvia–Estonia and Latvia–Lithuania.

The total received power of the 5G NR signal can be derived using the formula P_total_ = SS-RSRP + 10 × log10 (12 × N), where N is the number of RBs. After that, the recalculated P_total_ value can be compared with the appropriate frequency coordination threshold value given in Table 5. The table provides calculation results for the appropriate SS-RSRP values converted to electromagnetic field intensity for different channel bandwidths. In Table 5, green colour-highlighted results are those which do not exceed the frequency coordination threshold values from Table 4, and the blue colour-highlighted results exceed those values.

The study results show that the SS-RSRP level value −110 dBm (at the border between countries) is ensured, but the SS-RSRP level value −95 dBm is not ensured by the existing frequency coordination agreements between Latvia–Estonia and Latvia–Lithuania for the 700 MHz band. The SS-RSRP level values −110 dBm and −95 dBm (at the border between countries) are ensured by the existing frequency coordination agreement between Latvia–Estonia for the 3500 MHz band but are not ensured by the existing frequency coordination agreement between Latvia–Lithuania.

If the frequency coordination agreements of the 3500 MHz band concluded according to recommendation ECC/REC/(15)01 the field strength levels for the case of synchronized TDD networks, then the conditions for SS-RSRP, both −95 dBm and −110 dBm, are fulfilled. Thus, 5G NR TDD networks should use a common frame structure (uplink/downlink timeslot) and common time synchronization to ensure maximum electromagnetic compatibility and to reduce mutual interference between the communication networks of neighbouring countries.

### 4.4. The 5G BS Location Selection Principles and Criteria for Determining the Uncovered Areas

With regards to 5G BS location selection along the Via Baltica, Tallinn–Tartu–Valga, and Valga–Valka road routes, the existing passive infrastructure (e.g., BS towers, fibre optics, electricity lines) is planned to be used as much as possible. It is planned that 5G passive infrastructure along the Rail Baltica railway will be projected by the Rail Baltica company. For Lithuania, 5G BS location selection principles along Via Baltica are described in the study [5].

For the determination of uncovered-by-5G-BSs areas along the Via Baltica, Tallinn–Tartu–Valga, and Valga–Valka roads and the Rail Baltic railway, the appropriate 5G mobile network coverage calculations for Estonia and Latvia will be performed. For Lithuania, such calculations were performed in the study [5].

An examination of mobile service coverage for the existing network infrastructure of radio BS towers will be performed to determine “white areas”. Within the context of this study, “white areas” are defined as areas where the secondary synchronization reference signal receive power (SS-RSRP) level is lower than −110 dBm and −95 dBm (see Table 6).

### 4.5. Modelling of 5G Coverage along Transport Routes

For modelling 5G coverage along transport routes, available software will be used. VAS ES plan to use ATDI software “HTZ Communications”, but Telia and ELISA plan to use planning tool Forsk “Atoll”. For Lithuania, such calculations were performed within the study [5].

For the modelling of 5G networks coverage, using the parameters provided in Table 6 is considered. The data mainly come from interviews with stakeholders and consultations with the industry.

### 4.6. Cost Reduction Methods of 5G Corridors Development

In the previous sections, we studied the necessary infrastructure along the respective transport corridors, as well as the technical parameters of mobile communication networks that would meet the requirements of 5G transport corridors. Each phase of the infrastructure construction of the 5G transport corridors has variable costs. Since cost strongly influences any network planning, in this section, we will investigate methods to reduce these costs to develop 5G corridors cost-effectively. Furthermore, costs depend on local regulation; thus, coordination and synergy between development projects in the Baltic states is crucial.

Each technology cycle (2G, 3G, 4G, 5G) brings advantages (such as higher data rate, higher system capacity) for a certain period, but over time, these advantages are not enough for new technologies. Growing demand related to 5G use will trigger investment across all network domains—core networks, radio access networks, and the RF spectrum. 

A significant part of investment in 5G infrastructure must be addressed to power supply across transport corridors (including electric generators, batteries, renewable energy solutions, etc.) to guarantee continuous service. There must be very precise and thoughtful planning of the 5G network, including the structure of power supply, to avoid additional costs for new electricity connections to the infrastructure in the future.

The “radio access network infrastructure” and “Increased fiber optic roll out” sections of the passive infrastructure are applicable to the transport corridor of this study; therefore, within the framework of reducing the development costs of the 5G network, we will investigate these two aspects.

Before reporting the cost reduction methods, we start by reporting the expected cost of the different infrastructure elements.

#### 4.6.1. Cost of Upgrading or Adding New Sites 

In this section, the cost of upgrading or building new sites is broken down. The cost of the different elements is calculated based on the vendors’ pricing and operators’ experience. The average cost of erecting a passive part of a cell tower (a tower, a cabinet, a fence, an access road) is around EUR 156,000/site and EUR 397,700/site for Estonia and Latvia, respectively. The average cost of upgrading or installing active RAN equipment is around EUR 50,000/site for Estonia and Latvia. The average optical network connection cost per meter is EUR 40 and EUR 32 for Estonia and Latvia, respectively. The average power grid connection cost depends on the distance to the electric substation and the nature of the needed upgrades/improvements. For small distances that are usually less than 400 m (these types of distances are called amperage zones), the average cost is around EUR 5280 and EUR 8030 for Estonia and Latvia, respectively. On the other hand, the average cost for distances greater than 400 m reaches up to EUR 35,000. Finally, the average cost of the land is around EUR 15,000–30,000/site; however, the on-state properties do not have to pay rent.

#### 4.6.2. Reduction in the Costs Related to Radio access Network Infrastructure 

There are two modes of architecture for 5G network deployment:

- Nonstandalone, in which the 5G network is built and depends on the 4G network, specifically for control and coverage. Moreover, this leads to the absence of some of the 5G features, such as slicing.
- Standalone, in which the 5G network has its own equipment for its operation.

The cost of building standalone-mode 5G BSs far exceeds the costs of building 5G nonstandalone BSs. For example, the 5G nonstandalone architecture uses a 4G core network (Evolved Packet Core), while the 5G standalone architecture uses a completely new core (based on the Cloud Core with virtualized network functions). The cost depends on how large the network will be; an estimate of the minimum cost of a private 5G standalone core is EUR 500,000. Therefore, while the additional revenue potential of 5G is still unclear, such a gradual 5G implementation with a nonstandalone approach allows operators to reduce immediate investments. The MNOs of the Baltic states have also chosen the 5G nonstandalone mobile communication network implementation model.

Based on the above approach to the development of mobile communications networks in the Baltic states and because the investment project includes three separate project implementation territories as important cost reduction criteria, we can consider the factors described in the following subpoints.

**Figure 4:**
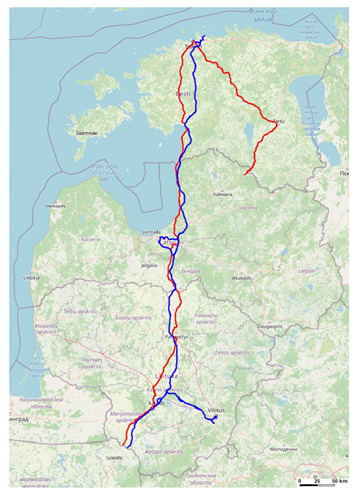
Road (red line) and railway (blue line) 5G corridors.

(a) Location of mobile communication towers (masts): There are several places where the Via Baltica highway and Rail Baltica railway line tracks overlap (see Figure 4). Therefore, when planning the development of mobile communication networks along 5G corridors, it could be necessary to consider the towers along the Via Baltica and the towers planned along the Rail Baltica railway line.

The construction of mobile communication towers (masts) in the protective zone of the 5G corridor where possible or on state-owned properties would reduce the land lease costs incurred by building communication towers on private land. Furthermore, the Law of Property Act, or “Asjaõigusseadus” [36], of Estonia must be considered. This law, among other things, regulates land use for networks which are in the public interest. Article 158 of the Act about technical networks and facilities necessary for public interests foresees, ”The owner of an immovable property is obliged to support a utility network or facility on his immovable property and to permit its construction on the immovable property if the utility network or facility is necessary in the public interest and there is no other technically and economically more feasible option for connecting the place of consumption of the person who wishes to connect to the utility network or facility to the utility network or facility or for the development of a utility network or facility“.

**Figure 5:**
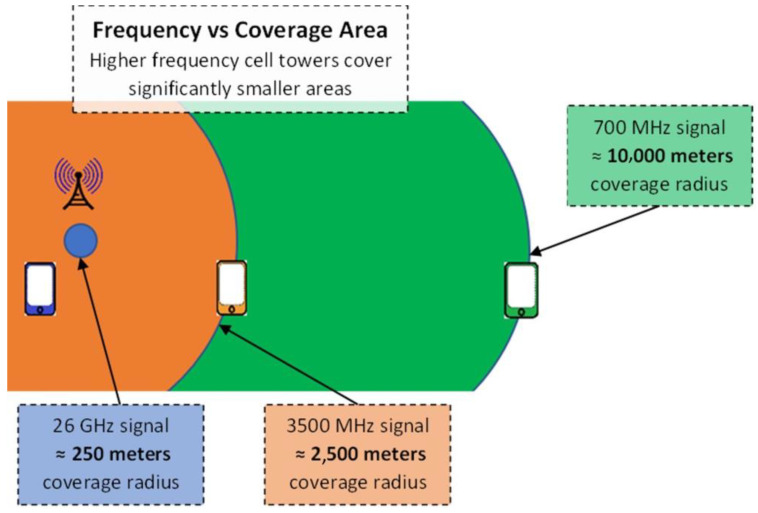
Frequency vs. coverage area—[38] modified.

(b) Communication tower infrastructure sharing between MNOs: According to the GSMA (Groupe Special Mobile Association) [37], from the historically compiled information on the study of network sharing cases, it can be concluded that mobile network sharing contributes to up to 50% savings and is expected to be slightly less than 40% of the total cost of a network using 5G technology. A significant part of the savings from the total costs can be obtained by sharing the passive infrastructure (including the sharing of mobile communication towers and power supply).
(c) Recommended use of the 700 MHz and 3500 MHz radio frequency bands: For physical reasons, lower frequencies (in our case, the 700 MHz frequency band) are more suitable for rural areas, because lower radio frequencies have a longer wavelength; therefore, the propagation of signals in free space will be better (see Figure 5). In addition, at higher radio frequencies (such as 3500 MHz, even more in the 26 GHz band), signals are more easily absorbed by obstacles. A typical cell tower operating in the 700 MHz frequency band can serve a user up to 10 km away. Therefore, using the 700 MHz radio frequency band for better coverage will require a smaller number of towers. In this way, the cost of building the towers as well as the cost of electricity is reduced.

On the other hand, when comparing the data transmission speeds in the 700 MHz and 3500 MHz radio frequency bands, it is important to note that the 3500 MHz frequency band offers extended bandwidth, which allows for a significantly higher data transmission speed and capacity. Therefore, it would be rational to use the 3500 MHz frequency range in densely populated areas.

It should be noted that with 5G expansion, technology such as dynamic spectrum sharing (DSS) allows for the utilization of 5G and 4G in the same frequency bands. One of the advantages of the DSS is that it is an essential enabler of the quick and cost-effective deployment of 5G networks with vast coverage areas using the existing low and mid bands of the spectrum. It allows for the use of legacy LTE hardware for 5G and reuse of LTE spectrum dynamic resource allocation between LTE and 5G. One disadvantage is that using DSS decreases LTE and NR networks’ capacity.

#### 4.6.3. Reduction in the Costs Related to the Increased Fiber Optic Network Roll Out

MNOs must conduct fibre cables installation operations on a large scale to improve connectivity and transmission. Fibre deployments are necessary for supporting urban areas’ small-cell deployment, in addition to assisting networks in meeting latency and capacity 5G requirements. Therefore, the creation of such an optical fibre infrastructure network is important and will be one of the cornerstones in the development of future 5G mobile communication networks. In the coming years, as part of the investment projects, the laying of optical fibre along the Via Baltica and Rail Baltica transport corridors will be implemented. The creation of such a common passive infrastructure network will allow for the development of mobile operators’ communication networks and significantly reduce costs for each stakeholder.

For now, in rural areas, one of the alternatives to optical fibre is the use of radio relay lines (microwave lines). Since the installation of such microwave lines is relatively quick and simple (no digging is required, as in the case of laying optics), the costs are significantly reduced. However, it should be noted that microwave lines technology is not as secure as optical fibre, and the microwave line signal is sensitive to weather conditions and interference. Furthermore, optical cables can provide more capacity, needed for 5G networks, and the required V2X service reliability. For these reasons, the use of microwave lines will decrease significantly in the coming years.

## 5. Phase 4—Network Planning

This section provides RF network modelling exercise results to provide uninterrupted 5G coverage to the Via Baltica and Rail Baltica transport corridors crossing Estonia and Latvia and on the border with Lithuania, as well as the Valka–Valga road (Latvia–Estonia), capable of cross-border 5G services. The network modelling results aim to foresee the number of new sites that need to be built on the said transport corridors, examining also the existing infrastructure for such purposes.

To prepare this section, the network planning task for transport corridors was executed, with four distinct scenarios. The first and second scenarios focus on coverage planning utilizing all currently available towers/masts/site locations considering the 700 MHz and 3500 MHz frequency bands. In the third and fourth scenarios, coverage planning extends beyond the existing masts and incorporates newly planned masts/towers. Coverage modelling adds a different number of new towers/masts for each route considered. In all four scenarios, network planning is conducted with a minimum signal level (SS-RSRP) threshold of −110 dBm and −95 dBm. All network planning has been carried out using specialized radio network planning software. Extensive analysis and mapping have been conducted to ensure optimal coverage along the transport corridors.

First, we start with the network planning for the Estonian side, followed by the network planning for the Latvian one.

### 5.1. Importance and Length of the Transport Corridors

This part addresses the length and importance of the transport corridors for which the RF network coverage plans will be provided. 

The total length of Via Baltica is 1722 km, of which the length of the Latvian section (Ainaži–Grenctāle) is 202 km. The Via Baltica road (E67) is a transport corridor that extends from the Czech Republic (Prague), crossing Poland, Lithuania, Latvia, and Estonia, to Finland, which and can be reached by ferry. Thus, Via Baltica connects the Baltic states with other important TEN-T routes and services more than 30,000 vehicles per day (referring to the statistics of SJSC Latvian State Roads [39]), being one of the major arteries for transit. In addition, Via Baltica is the only north–south transport corridor for freight transport between Lithuania, Latvia, and Estonia.

The length of the Tallinn–Ikla corridor in Estonia is 193 km. This road section is a part of the Via Baltica road, and its importance lies within providing connectivity between Estonia and neighbouring countries, fostering economic and cultural ties. It provides a direct link between the Baltic states, facilitating trade, tourism, and transportation across the region. According to the Estonian Transport Administration [40], on average, 10,000 vehicles per day use the Tallinn–Ikla road, with some sections having a traffic density of 35,000 vehicles per day. 

The length of the Tallinn–Tartu–Valga corridor in Estonia is 261 km. It connects two of the largest cities in Estonia, Tallinn and Tartu. The road facilitates the movement of goods, services, and commuters between these two important economic centres. The Tartu –Valga road provides an essential route for cross-border travel and trade, contributing to bilateral relations and economic cooperation between Estonia and Latvia. According to the Estonian Transport Administration [40], on average, 8100 vehicles per day use the Tallinn–Tartu–Valga road, with some sections having a traffic density of 27,800 vehicles per day.

The length of the Valka–Valga corridor in Latvia is 10 km. It is important to mention that this section of the road is not part of the Via Baltica road, but its importance is essential because it is the Latvian border crossing point of the TEN-T route Tallinn–Tartu–Valga in Valka. It is essential for local and transit freight. 

The length of the Rail Baltica corridor in Estonia is 213 km, in Latvia is 265 km, and in Lithuania is 392 km, and the total length of it in the Baltic states is 870 km. Rail Baltica is the most important project of the North Sea–Baltic Corridor of the TEN-T, which aims to integrate the Baltic states into a single European railway network. A two-way railway line is planned from Tallinn to the Lithuanian–Polish border, where it will further connect with a modernized railway line meeting the technical requirements of Rail Baltica to Warsaw, thus providing connections with the largest European ports—Hamburg, Rotterdam, and Antwerp.

### 5.2. Network Planning in Estonia

This section provides information on the network planning results in Estonia for the different transport corridors with existing infrastructure for 700 MHz and 3500 MHz coverage with minimum signal levels of −110 dBm and −95 dBm. After finding the coverage for the current infrastructure, new sites (infrastructure) are proposed to improve the coverage, and then the percentage of coverage is recalculated. The “Atoll” radio network planning software is used. The planning parameters are shown in Table 6. Furthermore, information about all available towers is gathered from the Estonian Land Board Buildings dataset [41].

The results are provided in Table 7. Generally, 700 MHz provides very good coverage for the −110 dBm minimum signal level and good coverage for −95 dBm. The addition of new sites further improves the coverage for −95 dBm. On the other hand, 3500 MHz does not seem to provide good coverage for the −95 dBm minimum signal level, even with the addition of new sites; hence, further investigation or workarounds are needed.

### 5.3. Network Planning in Latvia

Coverage assessment procedures, similar to those performed in Estonia, are performed for Latvia. However, for the Latvian transport corridors, the ATDI “HTZ Communications” radio network planning software is used. The modelling parameters are shown in Table 6. 

Rail Baltica performed the study “Radio Coverage Concept Design (D0101-SIE-ZZ-ZZ-REP_AP-O-00007)” (10 August 2022) (hereinafter—Concept Design) for the radiocommunications planning of the Rail Baltica railway route.

According to the document “BEREC Guidelines on Geographical surveys of network deployments. Verification of information”, a mobile broadband technology is considered to cover a pixel if 95% of the grid regions are served by the technology with a high probability of successful reception; this means that there is a 95% likelihood of service reception. Theoretically, this principle can be applied to a whole territory which consists of such pixels. A more relaxed territory coverage percentage value at a signal level of −95 dBm could be assumed at this 5G corridors development stage.

Furthermore, it is crucial to mention that Latvia’s coverage calculations for 3500 MHz frequency band additional modelling have been carried out for 50 MHz channel bandwidth, due to actual assigned radio frequency spectrum rights of use in the 3400–3800 MHz band in Latvia.

The results of the coverage analysis for the existing and new infrastructure are provided in Table 8. The results show a trend similar to the one observed on the Estonian side, i.e., Table 7. The table shows the coverage percentage when the desired received signal levels are −100 dBm and −95 dBm. From the table, it is clear that at higher signal levels, the coverage is more challenging. Also, the impact of adding new sites can be seen in the table. Note that for the same transmission power, the 50 MHz bandwidth provides better coverage than the 100 MHz bandwidth, as the power is distributed on less bandwidth. However, the smallest bandwidth will result in a lower data rate and capacity. In case a higher capacity is required, additional sites are needed, or more output power should be used, which is subject to regulations.

Simulation results could differ from the real-life situation, as the simulations are performed based on different assumptions in the study and parameters. For example, simulations demonstrate that clutter data may significantly affect coverage calculation results. Furthermore, the clutter data have been used theoretically, and no field research has been carried out within this exercise due to the study’s timeline and focus. Thus, the theoretical data might differ from the actual situation in the transport corridors.

### 5.4. Estimated Total Cost for New and Upgraded Sites

After identifying the needed new sites and upgrades, the total cost is reported in this section, according to the values presented in Section 4.6.1. Note that some predictions have been made in the cost calculations based on the expertise of the mobile network operators.

For the Estonian side, the total investment needed for all transport corridors studied in the Estonian territory is EUR 5,388,820 for the passive network components, EUR 13,888,820 including the active RAN parts in the case of one operator’s RAN and including all the new sites and upgrades of the existing sites, and EUR 30,888,820 in the case of the three operators’ RANs. To show the impact of the different elements presented in Section 4.6.1, we further investigate these obtained costs. The total cost of the passive parts of the towers for all the sites is EUR 3,276,000. As for the optical fibre network, the total cost is EUR 1,725,300. The total cost of power grid connection is EUR 387,520. The sum of the three values results in the reported EUR 5,388,820 passive network cost for building 21 new sites. The total cost, including the active RAN equipment, is EUR 6,438,820, which is approximately equivalent to EUR 306,610/site. On the other hand, the cost of utilizing the existing infrastructure and upgrading 97 sites is approximately EUR 4,850,000, which is equivalent to EUR 50,000/site. The Rail Baltica sites’ cost is EUR 2,600,000. The sum of these three values results in the reported EUR 13,888,820. From these costs, we note several points related to Section 4.6.1 and the study. The cost of building new sites is six times the cost of upgrading; this shows why it was crucial in this study to identify and utilize the existing infrastructure. Additionally, it justifies using different frequency bands, including 700 MHz, to increase coverage and reduce the cost of building new sites. 

For the Latvian side, the total investment needed for all transport corridors studied in Latvian territory is EUR 6,667,020 for the passive network components, EUR 20,067,020 including the active RAN parts in the case of one operator’s RAN and including all the new sites and upgrades of the existing sites, and EUR 46,867,020 in the case of the three operators’ RANs. The impact of utilizing the infrastructure and other elements is very similar to the one observed from the Estonian one. The cost for the new sites is approximately EUR 463,563/site. The cost of upgrading the sites is approximately EUR 50,000/site. 

### 5.5. Estimated Work Timeline for New Sites

Regarding the process of building a BS, ten types of permits or so-called authorizations are identified within the three Baltic states and are presented in Table 9. The information about each type of document necessary is depicted with the approximate timeframe necessary to obtain the document in each country where it applies and what kind of legal documents regulate such permits (if applicable).

It is crucial to mention that in Latvia the time to build a new BS may vary depending on the municipality where the BS is planned. The example in the table shows the average time required for a new BS. Based on their experience, the mobile operators state that on average it takes from 12 to 18 months to build a BS. But it is not uncommon for the process to be even longer. The main contributor to long base station development times is related to land questions. Finding a suitable land plot for a mobile network tower and negotiation time length with landowners is something that cannot be predicted precisely. It is possible that finding a suitable land plot could take many years.

## 6. Conclusions and Recommendations

In this work, we provided a detailed description of coverage and network planning procedures. Even though this work was performed for the Baltic corridor, it can serve as a guideline for other cross-border projects. 

In this study, we presented the steps for the 5G coverage planning of cross-border transport corridors, where coverage planning was carried out for rail and road corridors. Stakeholders are one of the main elements of coverage planning; hence, for this study, we interviewed the stakeholders, and then we started this work by introducing them and the main results of the interviews. Moreover, the information obtained from these interviews was used in the coverage planning that is performed in this work. Then, the binding documents between the countries across borders were presented to show the legal side of the process. Next, we presented the requirements, either performance, service continuity, infrastructure, or supervisory requirements. Additionally, the spectrum utilization in each country by the different MNOs was presented. 

An essential aspect of successful coverage planning is cost efficiency. Therefore, the key cost-consuming elements of coverage planning were identified. The main cost-consuming element is building new sites; thus, we started by identifying the existing infrastructure and the exact corridor points that needed to be covered. In addition to utilizing existing infrastructure, we proposed cost reduction methods. Finally, we used the parameters and requirements obtained from the stakeholders’ interviews to perform coverage planning and then presented the results of network planning. Coverage planning was performed using 700 MHz and 3500 MHz. The exact locations and number of new sites were found, and the total cost and cost per element were calculated, where it was found that the average cost of upgrading an existing site is six times less than the cost of building a new one. It was highlighted how important it is to identify and utilize existing infrastructure in addition to the smart utilization of the spectrum. In the final subsection, the estimated timeline was presented. 

From the results of this study, stakeholders’ interviews, and the conducted simulations, we recommend the following:

- As seen from the results, in order to provide −95 dBm signal level coverage along transport corridors in the 3500 MHz frequency band, a substantial expansion of tower infrastructure is necessary. To enhance 5G coverage and throughput for the corridor and reduce the required number of needed new towers, it would be beneficial to explore the utilization of frequencies beyond 700 MHz and 3500 MHz for the purpose of transport corridor coverage.
- In this study, as reported in the coverage modelling parameters, we used a digital map resolution of 50 × 50 m and clutter data. When planning 5G networks coverage along transport corridors (especially in the 3500 MHz frequency band), it is recommended to use higher-resolution-than-20 m digital elevation maps with accurate clutter data (if available), which would provide more precise simulations results.
- As reported in the Results section, parameters and assumptions used in coverage simulations (e.g., clutter data) should be verified with field measurements (if possible) along actual transport corridors and, in case of necessity, be adjusted accordingly.
- When determining the positioning of towers, it is advisable to prioritize the use of the 3500 MHz frequency for coverage calculations. This is because higher frequencies typically result in smaller coverage areas. By ensuring that coverage is achieved using the 3500 MHz frequency, it can be inferred that coverage will also be provided by lower frequency bands.

## Figures and Tables

**Table 1:** Important performance indicators [28].

Category	Type	4G	5G	Comments
Network	Coverage	99%	99% *	* Using, for example, DSS (dynamic spectrum sharing), 99% is theoretically possible; however, it is highly unlikely that any country will aim for 99% 5G coverage in the future.
	Cross-border	Yes	Yes	
	Availability	99%	99%	
Cross-border impact	Service continuity	No	Yes	
	Roaming duration	>1 min	0.5–1 s ^+^	^+^ Expected

**Table 2:** The 5G NR signal quality parameters.

	Condition	SS-SINR (dB)	SS-RSRQ (dB)	SS-RSRP (dBm)
RF Condition	Excellent	≥20	≥−10	≥−80
	Good	13 to 20	−10 to −15	−80 to −95
	Mid cell	0 to 13	−15 to −20	−95 to −110
	Cell edge	≤0	<−20	≤−110

**Table 3:** Frequency channel assignments [33].

V2X Operating Band	Sidelink (SL) Transmission Operating Band	Sidelink (SL) Reception Operating Band	Duplex Mode	Interface
	F_UL_low_–F_UL_high_	F_DL_low_–F_DL_high_		
n38 ^1^	2570–2620 MHz	2570–2620 MHz	HD	PC5
n47	5855–5925 MHz	5855–5925 MHz	HD	PC5
Note ^1^: When this band is used for V2X SL service, the band is exclusively used for NR V2X regions.				

**Table 4:** Frequency coordination threshold values (at the border for preferential PCIs) in force [35].

Frequency Band	LVA–EST	LVA–LTU
700 MHz	59 dBµV/m/5 MHz at border at 3 m (recalculated value: 62 dBµV/m/10 MHz at border at 3 m)	59 dBµV/m/5 MHz at border at 3 m (recalculated value: 62 dBµV/m/10 MHz at border at 3 m)
3500 MHz	79 dBµV/m/5 MHz at border at 3 m (for synchronized TDD networks) (recalculated values: 89 dBµV/m/50 MHz at border at 3 m or 92 dBµV/m/100 MHz at border at 3 m)	32 dBµV/m/5 MHz at border at 3 m (planned to review the agreement) (recalculated values: 42 dBµV/m/50 MHz at border at 3 m or 45 dBµV/m/100 MHz at border at 3 m)

**Table 5:** Calculation results.

Frequency Band	SS-RSRP Value	LVA–EST	LVA–LTU
		P_total_ Value Recalculated in dBµV/m Units (Assuming G_rx_ = 0 dBi)	
700 MHz (CH_BW_ = 10 MHz) (for SCS = 15 kHz)	−110 dBm	53 dBµV/m/10 MHz at border	53 dBµV/m/10 MHz at border
	−95 dBm	68 dBµV/m/10 MHz at border	68 dBµV/m/10 MHz at border
3500 MHz (CH_BW_ = 50 MHz) (for SCS = 30 kHz)	−110 dBm	70 dBµV/m/50 MHz at border	70 dBµV/m/50 MHz at border
	−95 dBm	85 dBµV/m/50 MHz at border	85 dBµV/m/50 MHz at border
3500 MHz (CH_BW_ = 100 MHz) (for SCS = 30 kHz)	−110 dBm	73 dBµV/m/100 MHz at border	73 dBµV/m/100 MHz at border
	−95 dBm	88 dBµV/m/100 MHz at border	88 dBµV/m/100 MHz at border

**Table 6:** The 5G coverage modelling parameters.

Parameters	Values
BS Parameters	
Frequency band	700 MHz (FDD); 3.5 GHz (TDD)
Channel bandwidth	For 700 MHz band: 10 MHz; for 3.5 GHz band: 100 MHz/50 MHz
BS height	For road routes: existing BS heights; and for new BSs: 66–84 m; for Rail Baltica route: 30 m (according to Rail Baltica data)
Antenna configuration	For roads: three sectors (for existing BS—current configuration); for Rail Baltica: two sectors
Antenna pattern	For 700 MHz band: typical antenna (e.g., H: 65 deg; V: 7 deg); for 3.5 GHz band: AAS with traffic beam envelope
Receiving SS-RSRP level (downlink)	Min. value: −110 dBm; upgrade value: −95 dBm
Cell radius limitation	Example values: for 700 MHz band: 5 km (up to 15 km); for 3.5 GHz band: 2.5 km (up to 4.6 km)
BS antenna type	For 700 MHz: passive; for 3.5 GHz: AAS
5G network type	SA
User Equipment Parameters	
Receiving antenna height	1.5 m
Antenna type	Omnidirectional
Antenna gain	0 dBi
Calculations Assumptions	
Propagation model	For 700 MHz: Okumura–Hata or other relevant model; for 3.5 GHz: Aster Propagation Model (for use in Forsk “Atoll”) or other relevant model
Digital map resolution	Min. resolution: 50 × 50 m
Clutter/building data	Yes/no

**Table 7:** Coverage simulation results for transport corridors of Estonia. EI is existing infrastructure, PI is planned infrastructure, and NS is new sites.

		Coverage [%]	
		Signal Level: −110 dBm	Signal Level: −95 dBm
Tallinn–Ikla	700 MHz (EI)	99.9%	96.5%
	700 MHz (EI + 8 NS)	99.9%	98.4%
	3500 MHz (EI)	97.2%	53.9%
	3500 MHz (EI + 8 NS)	99.9%	69.6%
Tallinn–Tartu–Valga	700 MHz (EI)	99.9%	96.3%
	700 MHz (EI + 13 NS)	99.9%	99.5%
	3500 MHz (EI)	94.6%	44.4%
	3500 MHz (EI + 13 NS)	99.9%	64.8%
Rail Baltica	700 MHz (EI)	93.9%	66.6%
	700 MHz (PI) Antenna height 30 m	100%	100%
	3500 MHz (EI)	79.9%	21.1%
	3500 MHz (PI) Antenna height 30 m	100%	93.4%
	3500 MHz (PI) Antenna height 45 m	100%	99.3%

**Table 8:** Coverage simulation results for transport corridors of Latvia. EI is existing infrastructure, PI is planned infrastructure, and NS is new sites.

		Coverage [%]	
		Signal Level: −110 dBm	Signal Level: −95 dBm
Valka–Valga	700 MHz (EI)	100%	98.0%
	700 MHz (EI + 1 NS)	100%	99.8%
	3500 MHz (EI) Bandwidth 50 MHz	92.1%	76.0%
	3500 MHz (EI) Bandwidth 100 MHz	89.6%	71.6%
	3500 MHz (EI + 1 NS) Bandwidth 50 MHz	99.3%	89.2%
	3500 MHz (EI + 1 NS) Bandwidth 100 MHz	98.3%	86.3%
Ainaži–Grenctāle (Via Baltica)	700 MHz (EI)	100%	96.7%
	700 MHz (EI + 15 NS)	100%	98.6%
	3500 MHz (EI) Bandwidth 50 MHz	85.3%	65.9%
	3500 MHz (EI) Bandwidth 100 MHz	80.8%	62.6%
	3500 MHz (EI + 15 NS) Bandwidth 50 MHz	93.3%	77.7%
	3500 MHz (EI + 15 NS) Bandwidth 100 MHz	90.1%	74.9%
Rail Baltica	700 MHz (EI)	99.9%	79.6%
	700 MHz (PI) Antenna height 30 m	100%	99.9%
	3500 MHz (EI) Bandwidth 50 MHz	61.3%	44.1%
	3500 MHz (EI) Bandwidth 100 MHz	56.7%	41.7%
	3500 MHz (PI) Bandwidth 50 MHz Antenna height 30 m	98.2%	91.1%
	3500 MHz (PI) Bandwidth 100 MHz Antenna height 30 m	97.1%	89.7%
	3500 MHz (PI) Bandwidth 50 MHz Antenna height 45 m	99.2%	94.8%
	3500 MHz (PI) Bandwidth 100 MHz Antenna height 45 m	98.4%	93.8%

**Table 9:** Process and timeline for the process of building a new BS in the Baltic states.

Process	Approx. Time in Months			Regulation Act EST	Regulation Act LVA	Regulation Act LTU
	EST	LV	LTU			
New site survey and agreement discussions with landowners	3–6	1–36	3–6			
Issuing of design criteria for new mast by local authorities	1	1	1	Building Code [42]		
If there is a need to change the zoning of some area (not needed everywhere; it depends on the local authorities and usually is needed in densely populated areas)	12	1	18–36	Planning Act [43]		
Design of mast	6	3–6	6–15			
Notarized agreement with the landowner	1	1–3	1			
Obtaining of building permits for local authorities	1	3–12	1	Building Code [42]	Construction Law [44]; Regulations of Minister Cabinet No. 501 [12]	Construction Law [45]
Power connection buildout	12	9–12	4–12			
After the building permit is issued, buildout of the site (includes ordering of the mast)	4	2–5	5–10			
Acceptance of electronic communication network installation project (needed if BS land or building is not owned by mobile operator)		0.5	-		Regulations of Minister Cabinet No. 501 [12]	
Authorization to use radio frequency assignment		1	-		Regulations of Minister Cabinet No. 133 [46]	
